# Supplementary material for: Performance Gaps and Optimization Strategies in Chinese Medical Large Language Models Based on MedBench: Evaluation Study
Source: JMIR AI. 2026 Jul 15;5:e86864. doi: 10.2196/86864 (PMC13372297; doi:10.2196/86864)
Supplement: Multimedia Appendix 1 [file ai-v5-e86864-s001.docx]

**Appendix A**

**Table S1.** Definition of assessment indicators

| **Indicator** | **Define** |
| --- | --- |
| Accuracy | Accuracy refers to the proportion of correctly answered question versions among all model predictions. Each multiple-choice question was presented five times, with the same content but with the correct answer systematically rotated across options A–E. An accuracy of 100% indicates that the model correctly answered all five versions of the question. |
| Robustness | The model's robustness refers to its ability to produce fully consistent answers when presented with the same choice question where options are randomly shuffled. |
| Macro-Recall | Macro-Recall calculates the recall based on each of the answer points designed in the quiz question separately, and then takes the average of these recalls for the score. |

**Table S2.** Incorrect Responses Labels and Definitions

| **Lable** | **Define** | **Mathematical Formulation** | **Illustrative Example** |
| --- | --- | --- | --- |
| Omissions | Model did not answer all points scored | Let *K*={k_1_, k_2_, ..., k_N_} be the set of key points required by the ground truth, and M be the set of key points present in the model's response. The Omission Rate (OR) is defined as: OR$=1-\frac{\vert K\cap M\vert}{\vert K\vert}$ | The prompt asks for three typical influenza symptoms (K={fever, cough, fatigue}). The model only outputs "fever and cough" (M={fever, cough}). Thus, OR=1−2/3=33.3%. |
| Hallucination | Model responses are not realistic or do not exist in the question stem | Let *E*_model_ be the set of medical entities or factual statements generated by the model, and *C* be the union of the provided context (question stem) and established medical knowledge bases (e.g., UMLS). The Hallucination Rate (HR) is defined as: $HR=\frac{{\vert E}_{model}\backslash C\vert}{\vert E_{model}\vert}$ | The provided patient history only mentions "hypertension." The model responds, "Considering the patient's history of hypertension and diabetes..." ("diabetes" is an ungrounded entity, belonging to *E*_model_ but not *C*). |
| Format Mismatch | Model does not output the content in the characteristic format as required or the output is in the wrong format | Let Φ be the set of formatting requirements (e.g., JSON structure, word/paragraph limits), and *L*(Φ) be the valid output space that satisfies these constraints. We define an indicator function I*_FM_*:  I*_FM_*$(Output) = \left\{ \begin{aligned} 0, &if Output\in L(\Phi) \\ 1, &if Output\notin L(\Phi) \end{aligned} \right.$ | The prompt strictly requires: "Output in JSON format, containing 'diagnosis' and 'treatment' keys." The model instead generates a plain text paragraph, failing to adhere to the structural constraint. |
| Causal Reasoning Deficiency | Over- or under-inference of the content of model responses | We model the reasoning process as a directed graph *G*=(*V*, *E*), where nodes V represent clinical states and edges E represent causal links. Let *E*^∗^ be the edges of the gold-standard reasoning chain and *Ê* be the edges generated by the model. The Deficiency Distance (*D_CR_*) is defined as: *D_CR_ = \|Ê\E*^∗^*\|+\|E*^∗^\*Ê\|* | Gold standard reasoning chain: Pathogen infection → Airway inflammation → Cough. Under-inference: "Cough is due to infection" (skipping the inflammation mechanism). Over-inference: "Infection → Malignant tumor" (introducing a non-existent causal link). |
| Contextual Inconsistency | Inconsistent or irrelevant model outputs | Let the model's response be a sequence of propositions *S*={s_1_, s_2_, ..., s_n_}. Inconsistency is triggered if there is an internal logical contradiction (i.e.,  ∃i,j such that s_i_∧s_j_ ⟹ False), or if the semantic similarity score *Sim*(*S*, *C*) between the response *S* and the context *C* falls below a predefined threshold τ (i.e., *Sim*(*S*, *C*) < τ). | In the first paragraph, the model states, "Aspirin is contraindicated for this patient," but in the concluding prescription, it writes, "Recommend taking Aspirin 100mg." The two propositions are mutually exclusive. |
| Unanswered | Model did not answer the question | Let Ω*_refusal_* be the set of common AI refusal templates, and H(Output) be the effective information entropy of the output. The indicator function I*_UN_* is defined as: $I_{UN} = \left\{ \begin{aligned} 1, &if Output\in\Omega refusal or H(Output)\approx0 \\ 0, &otherwise \end{aligned} \right.$ | The model outputs, "Sorry, as an AI, I cannot provide a medical diagnosis," or it simply generates meaningless punctuation marks. |
| Output Error | Model output is completely incorrect | Based on clinical facts, we define an accuracy scoring function *Score*_acc_(Output, Gold)∈[0, 1]. Given a lower bound of error tolerance ϵ, the Output Error indicator IOE is defined as: I*_OE_*$= \left\{ \begin{aligned} 1, &if Output<\epsilon\\ 0, &otherwise \end{aligned} \right.$ | The prompt asks, "What is the normal resting heart rate for adults?" The model answers, "10 to 20 beats per minute" (a statement that completely deviates from objective medical facts). |
| Deficiency in Medical Language Generation | The model's inability to translate everyday conversational content into clinically accurate medical text | Let *T_model_* be the set of symptom/disease vocabulary used in the model's response, and *V_standard_* be standard clinical terminologies (e.g., SNOMED CT, MeSH). The Non-professional Ratio (*R_np_*) is defined as: $R_{np}=\frac{{\vert E}_{model}\backslash C\vert}{\vert E_{model}\vert}$ | In a task requiring a professional discharge summary, the model uses colloquial terms like "tummy ache" and "runny nose" instead of standard medical terminologies like "abdominal pain" and "rhinorrhea". |

**Table S3.** Accuracy and Robustness Performance of Top10 Models

| Dimension | Number of question responses | Accuracy | Robustness |
| --- | --- | --- | --- |
| Medical Knowledge Question Answering | 3703 | 0.86 | 0.94 |
| Medical Language Generation | 1530 | 0.76 | / |
| Medical Language Understanding | 5784 | 0.71 | / |
| Complex Medical Reasoning | 891 | 0.72 | / |
| Healthcare Safety and Ethics | 32802 | 0.80 | 0.79 |
| Total | 44711 | / | / |


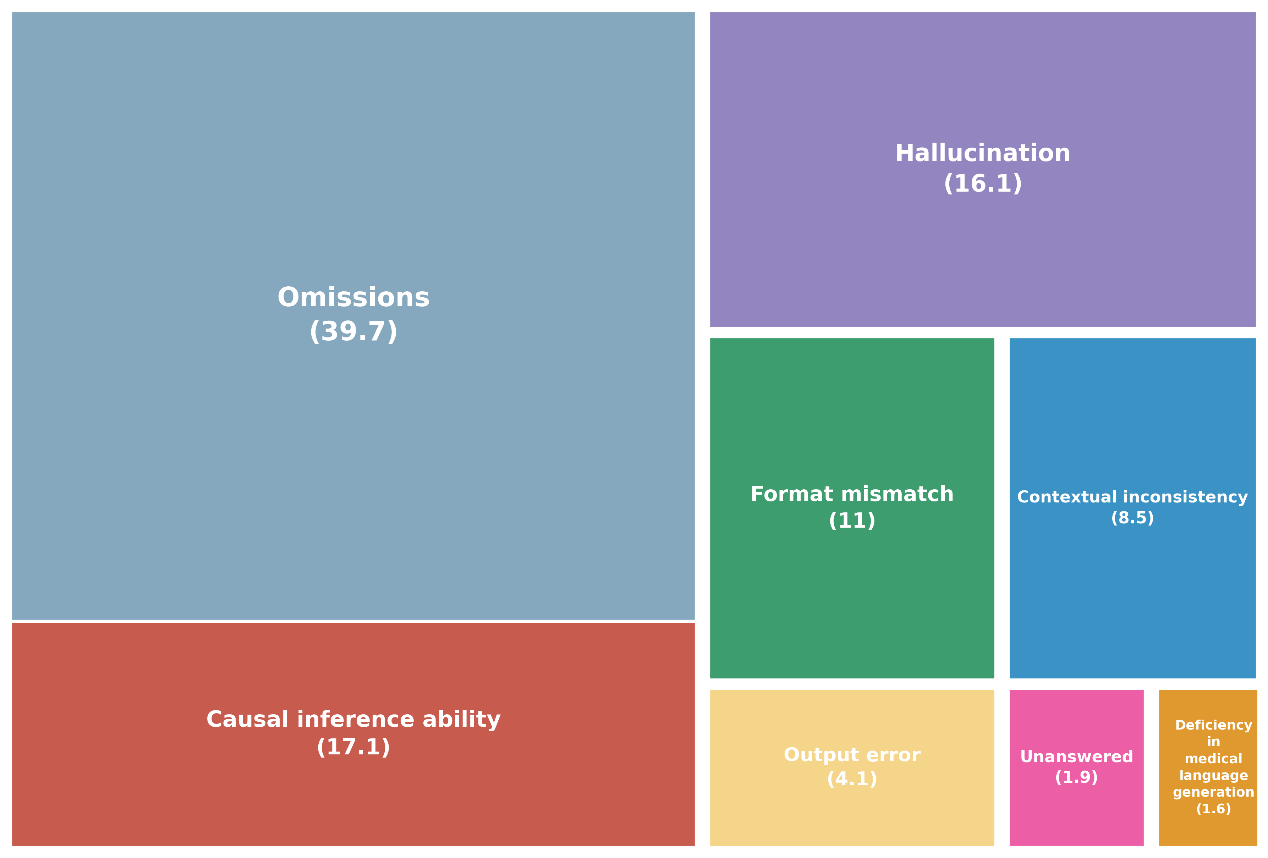


**Figure S1.** Proportions of incorrect responses in labels


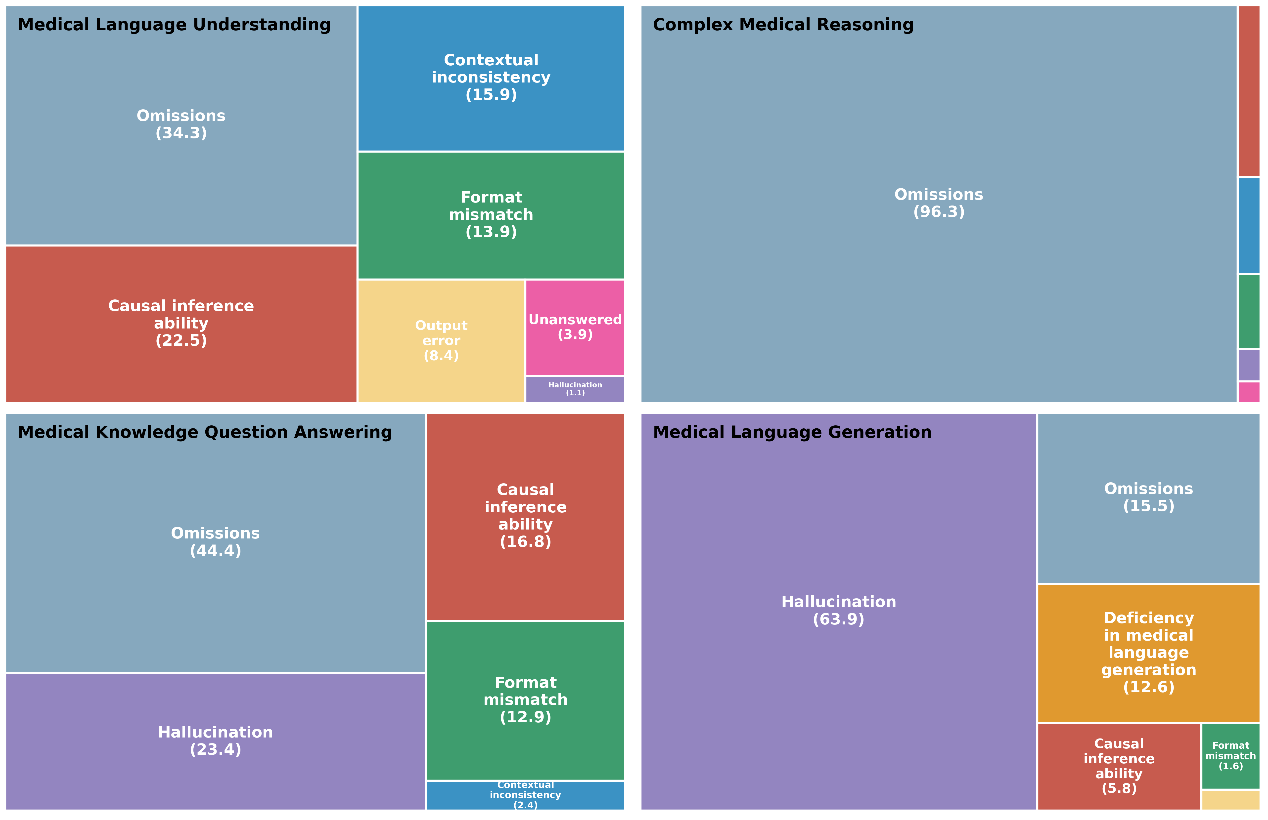


**Figure S2.** Proportions of incorrect responses in labels under different dimensions

**Table S4.** Labeling of incorrect responses and corresponding examples

**4.1 Omissions**

| **Question** | **Answer Points** | **Model Response** |
| --- | --- | --- |
| 肠结核有哪些临床表现？  What are the clinical manifestations of intestinal tuberculosis? | ["腹痛", "大便习惯改变", "腹部肿块", "全身症状", "低热", "盗汗", "贫血"]  ["abdominal pain", "change in bowel habits", "abdominal mass", "systemic symptoms", "low-grade fever", "night sweats", "anemia"] | 肠结核的临床表现主要包括：发热、盗汗、消瘦、腹痛、腹泻、便秘、腹部包块等。其中，腹痛多位于右下腹或脐周，呈隐痛或钝痛，餐后加重，排便后减轻。腹泻与便秘可交替出现，腹泻多为糊状便，无脓血。腹部包块多位于右下腹，质地较硬，活动度差，有压痛。部分患者可有肠梗阻表现，如腹胀、呕吐等。  The clinical manifestations of intestinal tuberculosis mainly include: fever, night sweats, emaciation, abdominal pain, diarrhea, constipation, abdominal mass, etc. Among them, abdominal pain is mostly located in the right lower abdomen or around the umbilicus, which is hidden pain or dull pain, aggravated after meals and alleviated after defecation. Diarrhea and constipation may occur alternately, and the diarrhea is mostly paste-like stool without pus and blood. Abdominal mass is mostly located in the right lower abdomen, hard texture, poor mobility, with pressure pain. Some patients may have intestinal obstruction manifestations, such as abdominal distension and vomiting. |

**4.2 Hallucination**

| **Question** | **Answer Points** | **Model Response** |
| --- | --- | --- |
| 粒细胞缺乏症的定义是什么?  What is the definition of granulocyte deficiency? | ["中性粒细胞绝对计数", "0.5 ×10^9/L"]  ["Absolute neutrophil count", "0.5 × 10^9/L"] | 粒细胞缺乏症是一种严重的血液疾病，主要特征是血液中的中性粒细胞数量极度减少或完全缺失。中性粒细胞是白细胞的一种，主要负责抵抗感染。因此，粒细胞缺乏症患者极易发生严重的感染，病情危重，死亡率高。  粒细胞缺乏症的发生可能与多种因素有关，包括粒细胞生成减少、成熟障碍、消耗过多等。一些疾病，如再生障碍性贫血、急性白血病、感染、肿瘤骨髓浸润等，可能导致粒细胞减少症。维生素B12或叶酸缺乏也可能导致粒细胞成熟障碍。此外，系统性红斑狼疮、败血症、严重感染等可能导致粒细胞过度消耗。  粒细胞缺乏症的治疗主要以升白细胞药物为主，同时需要控制感染。肾上腺皮质激素、升白细胞药物、粒细胞输注等方法也可以用于缓解症状。患者需要注意休息，保持局部卫生，避免感染，并避免食用辛辣刺激性食物。  Granulocyte deficiency is a serious blood disorder characterized by an extreme decrease or complete absence of neutrophils in the blood. Neutrophils are a type of white blood cell that is primarily responsible for fighting off infections. Therefore, patients with granulocyte deficiency are highly susceptible to serious infections, critical conditions and high mortality rates.  The development of granulocyte deficiency may be associated with a number of factors, including decreased production of granulocytes, impaired maturation, and excessive depletion. Some diseases, such as aplastic anemia, acute leukemia, infections, and bone marrow infiltration of tumors, may lead to granulocytopenia. Vitamin B12 or folate deficiency may also lead to impaired granulocyte maturation. In addition, systemic lupus erythematosus, sepsis, and severe infections may lead to excessive granulocyte depletion.  Treatment of granulocyte deficiency is primarily based on leukocyte-boosting drugs, and infection control is required. Adrenocorticotropic hormone, leukocyte-boosting drugs, and granulocyte infusion can also be used to relieve symptoms. Patients need to pay attention to rest, maintain local hygiene, avoid infection, and avoid spicy and irritating foods. |

**4.3 Format Mismatch**

| **Question** | **Answer Points** | **Model Response** |
| --- | --- | --- |
| 医学命名实体识别任务：请从给定医学文本中，识别出命名实体。  实体识别的格式为：  药物：""  医疗设备：""  医院科室：""  微生物类：""  身体部位：""  医疗程序：""  医学检验项目：""  症状和体征：""  疾病：""  Medical Named Entity Recognition Task: Please identify named entities from a given medical text.  The format for entity identification is:  Drug: ""  Medical device: ""  Hospital department: ""  Microorganisms: ""  Body part: ""  Medical Procedures: ""  Medical Test Programs: ""  Signs and symptoms: ""  Diseases: "" | "药物": []  "医疗设备": []  "医院科室": []  "微生物类": []  "身体部位": []  "医疗程序": []  "医学检验项目": []  "症状和体征": ["FMR-1基因的突变"]  "疾病": ["脆性X综合征"]  "Drugs": []  "Medical devices": []  "Hospital units": []  "Microbiology": []  “Body parts": []  "Medical procedures": []  "Medical tests": []  "Symptoms and signs": ["Mutations in the FMR-1 gene"]  “Diseases": ["Fragile X syndrome"] | 药物：  医疗设备：  医院科室：  微生物类：  身体部位：  医疗程序：  医学检验项目：  症状和体征：  疾病：脆性X综合征  基因：FMR-1基因  Drugs:  Medical Equipment:  Hospital Departments:  Microbiology:  Body Parts:  Medical Procedures:  Medical Test Program:  Signs and symptoms:  Disease: fragile X syndrome  Gene: FMR-1 gene |

**4.4 Causal Reasoning Deficiency**

| **Question** | **Answer Points** | **Model Response** |
| --- | --- | --- |
| 现病史  病史摘要  赵某，男，7岁，3天前无明显诱因出现腹部疼痛，伴寒战高热，最高体温38.7℃，于当地行“退热止痛”治疗后未见明显好转。患儿于1天前出现皮肤黄染，同时伴巩膜黄染，精神差，营养状况较差。病儿自发病以来，精神睡眠差，饮食差，大便成形，偶呈陶土样色，尿呈浓茶样色。  主诉  腹痛高热3天，伴皮肤黄染1天。  体格检查  T38.5℃，P92次/分，R22次/分，Bp110/78mmHg。  发育正常，营养中等，自主体位，查体合作，全身皮肤及巩膜黄染，上肢皮肤可见散在皮下出血点，全身浅表未及肿大淋巴结。两肺呼吸音粗，未闻及干湿啰音。心率92次/分，律齐，未闻及病理性杂音。腹部略膨隆，肝脏肿大，表面光滑，质地坚硬，边缘圆钝，于肝区肋弓下可触及一肿物，形状规则，大小约3cm×2cm，右上腹压痛，反跳痛（±），全腹叩诊鼓音，移动性浊音阴性，肠鸣可闻及。  辅助检查  实验室检查  血常规：WBC15.91×10^9/L，NEU89.7%，RBC3.74×10^12/L，Hb115g/L，PLT286×10^9/L。  肝功能：ALT70U/L，AST56U/L；总胆红素176μmol/L，间接胆红素25μmol/L，直接胆红素151μmol/L；碱性磷酸酶984U/L；谷氨酰转移酶1065.5U/L。  影像学表现  腹部CT平扫  1.CT检查显示胆总管呈巨大囊样扩张。  2.胆囊体积增大。  3.无肝内胆管远端分支扩张。  简述本例病人的诊断及诊断依据。  Present Medical History  (1) Summary of history  Zhao Mou, male, 7 years old, had abdominal pain with chills and high fever with a maximum temperature of 38.7℃ 3 days ago without any obvious cause, and did not show significant improvement after local treatment of “antipyretic and analgesic”. The child developed skin yellowing 1 day ago, accompanied by scleral yellowing, poor mental health and poor nutritional status. Since the onset of the disease, the child had poor mental sleep, poor diet, formed stools, occasionally clay-like color, and urine was strong tea-like color.  (2) Complaints  Abdominal pain and high fever for 3 days, accompanied by skin yellowing for 1 day.  Physical examination  T38.5℃, P92 beats/min, R22 beats/min, Bp110/78mmHg.  Development is normal, nutrition is moderate, autonomous position, cooperation in physical examination, generalized skin and sclera yellow staining, scattered subcutaneous hemorrhages can be seen on the skin of the upper limbs, generalized superficial not found enlarged lymph nodes. Respiratory sounds in both lungs were coarse, and no dry or wet rales were detected. Heart rate was 92 beats/minute, rhythmic, no pathological murmur was heard. The abdomen was slightly distended, the liver was enlarged, smooth surface, hard texture, rounded edges, a mass was palpable under the costal arch of the liver area, regular shape, size about 3cm×2cm, pressure pain in the right epigastric region, rebound pain (±), tympanic sound on percussion in the whole abdomen, mobile turbid sound was negative, and intestinal tinnitus could be heard.  Auxiliary examination  Laboratory examination  Blood routine: WBC15.91×10^9/L, NEU89.7%, RBC3.74×10^12/L, Hb115g/L, PLT286×10^9/L.  Liver function: ALT70U/L, AST56U/L; total bilirubin 176μmol/L, indirect bilirubin 25μmol/L, direct bilirubin 151μmol/L; alkaline phosphatase 984U/L; glutamyl transferase 1065.5U/L.  Imaging manifestations  Abdominal CT plain scan  1. CT examination showed a huge cystic dilatation of the common bile duct.  2. Increased gallbladder volume.  3. No dilatation of the distal branches of the intrahepatic bile duct.  Briefly describe the diagnosis and diagnostic basis of this patient. | ["先天性胆管扩张症", "腹部疼痛", "高热", "寒战", "黄染", "直接胆红素", "囊样扩张"]  ["congenital choledocholithiasis", "abdominal pain", "high fever", "chills", "yellow stain", "direct bilirubin", "cystic dilatation"] | 本例患儿的诊断可能为胆道梗阻导致的阻塞性黄疸，具体原因需进一步明确。  诊断依据包括：  1. 临床症状：患儿出现腹痛、寒战、高热，随后出现皮肤和巩膜黄染，精神差，营养状况较差。这些症状提示可能存在感染和/或肝脏疾病。  2. 体格检查：皮肤和巩膜黄染，上肢皮下出血点，肝脏肿大，质地坚硬，右上腹压痛，这些体征进一步支持肝脏疾病和/或胆道问题。  3. 实验室检查：血常规显示白细胞升高，中性粒细胞比例增高，提示有感染的可能。肝功能异常，ALT和AST轻度升高，总胆红素和直接胆红素显著升高，碱性磷酸酶和谷氨酰转移酶显著升高，这些都是胆道梗阻和肝细胞损伤的标志。  4. 影像学检查：腹部CT平扫显示胆总管呈巨大囊样扩张，胆囊体积增大，但无肝内胆管远端分支扩张。这表明胆道系统存在异常，可能是胆道梗阻的直接证据。  综合上述临床表现、体格检查、实验室检查和影像学检查，可以初步诊断为胆道梗阻导致的阻塞性黄疸。但是，需要进一步的检查，如胆道超声、MRCP（磁共振胰胆管造影）或ERCP（内镜逆行胰胆管造影）等，以确定梗阻的具体位置和原因，可能是胆结石、胆道炎症、先天性胆道异常或其他原因所致。  The diagnosis of this child may be obstructive jaundice due to biliary obstruction, the exact cause of which requires further clarification.  The diagnostic basis includes:  1. Clinical symptoms: the child presented with abdominal pain, chills, and high fever, followed by yellowing of the skin and sclera, poor mental health, and poor nutritional status. These symptoms are suggestive of possible infection and/or liver disease.  2. Physical examination: yellowing of the skin and sclera, subcutaneous hemorrhagic spots on the upper extremities, an enlarged, firm liver, and right upper abdominal tenderness are signs that further support the presence of liver disease and/or biliary tract problems.  3. Laboratory tests: Blood counts show elevated leukocytes and an increased percentage of neutrophils, suggesting infection. Liver function is abnormal with mild elevations of ALT and AST, significant elevations of total and direct bilirubin, and significant elevations of alkaline phosphatase and glutamyltransferase, all of which are markers of biliary obstruction and hepatocellular injury.  4. Imaging: CT scan of the abdomen shows a huge cystic dilatation of the common bile duct and an increase in gallbladder volume without dilatation of the distal branches of the intrahepatic bile ducts. This indicates an abnormality in the biliary system and may be direct evidence of biliary obstruction.  Taking into account the above clinical manifestations, physical examination, laboratory tests and imaging studies, a preliminary diagnosis of obstructive jaundice due to biliary obstruction can be made. However, further investigations such as biliary ultrasound, MRCP (magnetic resonance cholangiopancreatography) or ERCP (endoscopic retrograde cholangiopancreatography) are needed to determine the exact location and cause of the obstruction, which may be due to gallstones, biliary inflammation, congenital biliary anomalies or other causes. |

**4.5 Contextual Inconsistency**

| **Question** | **Answer Points** | **Model Response** |
| --- | --- | --- |
| 现病史  病史摘要  陈XX，男，65岁，近一周来反复排暗红色血便，伴头晕乏力。半天前再次排血便，量约600ml，并出现神志淡漠，尿量减少，12小时尿量为200ml。无发热、恶心、呕吐，既往病史无特殊。  主诉  反复排血便1周，加重伴少尿半天。  体格检查  生命体征T36.8℃，P105次/分，R20次/分，Bp90/60mmHg。体重60kg。  自主体位，神志淡漠，呼之可对答。全身皮肤及巩膜较苍白，四肢偏凉。两肺呼吸音清晰，未闻及干湿啰音。心率105次/分，律齐，未闻及病理性杂音。腹部平软，肝脾不大，肠鸣音活跃。双肾区无叩痛，双下肢无水肿。  辅助检查  血液分析：白细胞计数8.8×10^9/L，中性粒细胞比例0.7，红细胞计数2.8×10^12/L，血红蛋白78g/L  尿液分析：尿比重1.030，尿白细胞（-），尿潜血（-），尿蛋白（-）  大便潜血：（+），胃潜血：（-）  生化电解质：钾4.5mmol/L，钠138mmol/L，氯103.5mmol/L，二氧化碳结合力22mmol/L  肝肾功能：尿素氮9.8mmol/L，肌酐201µmol/L，谷丙转氨酶10U/L，谷草转氨酶20U/L  纤维结肠镜检查：提示降结肠及直肠可见弥漫出血点。  B超检查：肝、胆、脾、胰及泌尿系B超无特殊。  简述本例病人的治疗原则。  Present Medical History  Summary of history  Chen XX, male, 65 years old, had repeated dark red blood stools with dizziness and fatigue in the past week. Half a day ago, he passed blood stool again, with a volume of about 600 ml, and appeared to be apathetic, with a decreased urine output of 200 ml in 12 h. There was no fever, nausea, or vomiting, and his past medical history was unremarkable.  Complaints  Recurrent bloody stools for 1 week, aggravated with oliguria for half a day.  Physical examination  Vital signs T36.8℃, P105 beats/min, R20 beats/min, Bp90/60mmHg. weight 60kg.  He was in voluntary position, apathetic, and could answer when called. The skin and sclera were pale, and the limbs were cold. Breath sounds of both lungs were clear, and no dry or wet rales were heard. Heart rate was 105 beats/minute, rhythmic, no pathological murmur was heard. The abdomen was flat and soft, the liver and spleen were not large, and bowel sounds were active. There was no tenderness in both kidneys and no edema in both lower limbs.  Auxiliary examination  Blood analysis: white blood cell count 8.8×10^9/L, neutrophil ratio 0.7, red blood cell count 2.8×10^12/L, hemoglobin 78g/L.  Urinalysis: urine specific gravity 1.030, urine leukocytes (-), urine occult blood (-), urine protein (-)  Fecal occult blood: (+), gastric occult blood: (-)  Biochemical electrolytes: potassium 4.5 mmol/L, sodium 138 mmol/L, chloride 103.5 mmol/L, carbon dioxide binding capacity 22 mmol/L  Liver and kidney function: urea nitrogen 9.8 mmol/L, creatinine 201 µmol/L, alanine aminotransferase 10 U/L, azelaic aminotransferase 20 U/L  Fiber colonoscopy: suggesting diffuse bleeding spots visible in the descending colon and rectum.  Ultrasound examination: liver, gallbladder, spleen, pancreas and urinary tract ultrasound is not special.  Briefly describe the principles of treatment for this patient. | ["止血", "输血", "液体平衡", "电解质及酸碱平衡"]  ["Hemostasis", "Transfusion", "Fluid balance", “Electrolyte and acid-base balance"] | 1.该病例出血原因考虑为结肠病变，反复出血考虑为慢性失血，半天前大量出血为慢性失血急性加重。病人体重偏轻，平时饮食习惯不佳，半天前大量出血后尿量减少，考虑血容量不足，急性肾损伤。根据病人的血红蛋白水平及结肠镜检查结果，应予以输血补液，纠正贫血，维持水电解质平衡，同时请肛肠外科会诊讨论是否手术治疗。  2.在补充血容量后，病人尿量增加，肌酐可缓慢下降至正常。若肌酐继续上升，考虑行肾脏替代治疗。  3.病人的贫血主要原因为消化道出血，为缺铁性贫血，待病人病情稳定后，可予以补充铁剂。  1. The cause of bleeding in this case is considered to be colonic lesions, repeated bleeding is considered to be chronic blood loss, and massive bleeding half a day ago is an acute exacerbation of chronic blood loss. The patient's light weight, poor dietary habits in general, and decreased urine output after massive bleeding half a day ago are considered to be hypovolemia and acute kidney injury. According to the patient's hemoglobin level and colonoscopy results, blood transfusion and fluid replacement should be given to correct anemia and maintain the water-electrolyte balance, and at the same time, we should ask the anorectal surgery department to consult and discuss whether surgical treatment.  2. After supplementing blood volume, the patient's urine output increases, and creatinine may slowly decrease to normal. If creatinine continues to rise, consider renal replacement therapy.  3. The patient's anemia is mainly caused by gastrointestinal bleeding, which is iron deficiency anemia. After the patient's condition is stabilized, iron supplements can be given. |

**4.6 Unanswered**

| **Question** | **Answer Points** | **Model Response** |
| --- | --- | --- |
| 现病史  病史摘要  病人2017年8月15日夜间驾驶摩托车被出租车横向撞伤，当时诉腹部胀痛、左膝疼痛，急症入六安市人民医院，给予保守治疗腹痛未见明显好转，且腹痛加重。2017年8月17日转至安医大二附院，检查提示腹腔积液明显，急诊剖腹探查。术中见腹盆腔大量肠液样液体，约2000ml，全小肠肠壁炎症水肿，距屈氏韧带约45cm处小肠系膜撕裂，致约40cm小肠发黑，并可见2处破裂口。行小肠部分切除肠吻合+小肠结肠浆肌层修补术。术后病人持续高热，最高40摄氏度，低血压、乳酸持续性升高，最高7.66mmol/L，转至ICU给予呼吸机辅助呼吸、血管活性药物等治疗，病人未见明显好转。于2017年8月21日夜间8时转入我院，入院时病人神志淡漠，呼吸机辅助呼吸，心率增快，约130次/分；血压130/80mmHg左右；体温38.3℃，给予拔除腔静脉导管；抽取血培养，亚安培南+伏立康唑抗感染；给予抑酸、抑酶，静脉营养支持；监测膀胱压；病人全身组织水肿明显，限制液体输入，给予蛋白后利尿（24小时共出尿液约7000ml）。  主诉  车祸外伤术后多脏器功能障碍3天。  体格检查  （1）一般情况  神志模糊，精神欠佳，慢性病容，表情痛苦，发育正常，营养中等，匀称，抬入病房，被动体位，查体合作，语言正常，声音低微，对答切题。  （2）专科情况  腹部膨隆，腹式呼吸存在，无腹壁静脉曲张，未见肠型及蠕动波，无压痛及反跳痛，未触及包块。腹部正中可见一手术伤口，少量脓性液体，腹部可见右侧两根腹腔引流管。肝脾肋下未触及，未触及胆囊，Murphy征阳性。腹部鼓音区未及，无移动性浊音。肝上界位于右锁骨中线第5肋间，下界位于右季肋下缘，肝区无叩痛，脾浊音区正常，胆囊区无叩痛。肠鸣音弱，1次/分，未闻及血管杂音及摩擦音。  辅助检查  （1）实验室检查  白细胞计数9.5×10^9/L，红细胞计数3.28×10^12/L，血红蛋白98g/L，血小板计数498×10^9/L，C反应蛋白74.9mg/L。  （2）X线检查  床边胸片提示两肺炎症，左侧胸腔少量积液。  （3）CT检查  提示腹腔广泛渗出。  病情变化  2017年8月22日19时体温升高，最高39℃，20时血压下降，最低77/34mmHg；心率升高，最高140次/分。给予加快输液速度，补充晶体液；同时给予白蛋白、红悬、血浆等补充胶体液；给予冷沉淀改善凝血功能；22时病人血压恢复至120/80mmHg左右，心率恢复至80次/分。  该病人可下哪些诊断？  Present Medical History  Summary of medical history  The patient was driving a motorcycle at night on August 15, 2017 and was hit by a cab horizontally, at that time, he complained of abdominal distension and pain, left knee pain, and was admitted to the Lu'an People's Hospital in an emergency, and was given conservative treatment for abdominal pain which did not show any significant improvement and worsening of the abdominal pain.He was referred to the Second Affiliated Hospital of the Anhui University of Medical Science and Technology on August 17, 2017, and his examination suggested that there was obvious accumulation of fluid in the abdominal cavity, and he was urgently admitted to the hospital by caesarean section. Intraoperatively, a large amount of intestinal fluid-like fluid was seen in the abdominopelvic cavity, about 2,000 ml, inflammation and edema of the intestinal wall of the whole small intestine, and the mesenteric laceration of the small intestine about 45 cm from the flexural ligament, which resulted in the blackness of the small intestine of about 40 cm and 2 rupture orifices were seen. Partial resection of small bowel with intestinal anastomosis + small bowel colonic plasma muscle layer repair was performed. Postoperatively, the patient had persistent high fever up to 40 degrees Celsius, hypotension, persistent elevation of lactate up to 7.66 mmol/L. He was transferred to ICU and given ventilator-assisted respiration, vasoactive drugs, etc., and the patient did not show significant improvement. Transferred to our hospital at 8:00 p.m. on the night of August 21, 2017, at the time of admission, the patient was apathetic, ventilator-assisted respiration, heart rate increased, about 130 beats/min; blood pressure about 130/80 mmHg; temperature 38.3 ℃, given to remove the vena cava catheter; blood culture was taken, subamphetamine + voriconazole anti-infective; acid suppression, enzyme suppression, intravenous nutritional support was given; bladder pressure was monitored; the patient's systemic tissue Edema was obvious, fluid intake was restricted, and post-protein diuresis was given (a total of about 7000 ml of urine was produced in 24 hours).  Main complaint  Multi-organ dysfunction for 3 days after car accident trauma.  Physical examination  (1) General condition  Ambiguous, mentally ill, chronic look, painful expression, normal development, moderate nutrition, well-proportioned, carried into the ward, passive position, cooperative in body checking, normal speech, low voice, tangential answers.  (2) Specialized conditions  The abdomen was distended, abdominal respiration was present, no abdominal wall varices, no bowel pattern or peristaltic wave was seen, no pressure pain or rebound pain, and no mass was palpable. A surgical wound with a small amount of purulent fluid was seen in the middle of the abdomen, and two abdominal drains on the right side were seen in the abdomen. Liver and spleen were not palpated subcostally, gallbladder was not palpated and Murphy's sign was positive. Abdominal tympanic area was not reached and there was no mobile turbidity. The upper border of the liver was located at the 5th intercostal space in the right midclavicular line and the lower border was located at the lower border of the right quaternary ribs; there was no percussion pain in the hepatic region, the splenic turbid zone was normal, and there was no percussion pain in the gallbladder region. Bowel sounds were weak, 1 beats/minute, no vascular murmur or friction sounds were heard.  Auxiliary examination  (1) Laboratory tests  White blood cell count 9.5×10^9/L, red blood cell count 3.28×10^12/L, hemoglobin 98g/L, platelet count 498×10^9/L, C-reactive protein 74.9mg/L.  (2) X-ray examination  Bedside chest radiograph suggested inflammation in both lungs and a small amount of fluid in the left pleural cavity.  (3) CT examination  Suggests extensive exudation in the abdominal cavity.  Disease change  At 19:00 on August 22, 2017, the body temperature increased, with a maximum of 39°C. At 20:00, the blood pressure decreased, with a minimum of 77/34 mmHg; the heart rate increased, with a maximum of 140 beats/min. Accelerated infusion rate was given to replenish crystalloid; albumin, red suspension, and plasma were also given to replenish colloid fluids; cold precipitation was given to improve coagulation; at 2200 hours, the patient's blood pressure recovered to about 120/80 mmHg, and the heart rate recovered to 80 beats/min.  What diagnosis can be made in this patient? | ["腹腔感染", "脓毒症", "多脏器功能障碍"]  ["abdominal infection", "sepsis", "multiple organ dysfunction"] |  |

**4.7 Output Error**

| **Question** | **Answer Points** | **Model Response** |
| --- | --- | --- |
| 任务：将给定实体进行归一化，从候选的若干个ICD-10诊断标准词中选择出与原诊断描述匹配的词。  输入医学短语：  右侧睾丸胚胎癌  候选标准词：  甲状腺恶性肿瘤##癌##肺继发恶性肿瘤##恶性肿瘤放疗##转移性癌  子宫颈恶性肿瘤##鳞状细胞癌##高血压  麦克尔憩室##小肠憩室炎  抑郁发作##脑血管病  颊部血管畸形  睾丸恶性肿瘤##胚胎性癌  归一化术语：  TASK: Normalize a given entity to select from a candidate number of ICD-10 diagnostic criteria words that match the original diagnostic description.  Enter the medical phrase:  Embryonal carcinoma of the right testis  Candidate Criteria Words:  Thyroid malignancy##Cancer##Secondary malignant tumor of lung##Radiotherapy for malignancy##Metastatic cancer  malignant tumor of the cervix##squamous cell carcinoma##hypertension  Michael's diverticulum##Small bowel diverticulitis  Depressive episodes ##Cerebrovascular disease  Buccal vascular malformations  Testicular malignancy ##embryonic carcinoma  Normalized Terminology: | 睾丸恶性肿瘤##胚胎性癌  Testicular malignant tumors##Embryonal carcinoma | 鳞状细胞癌##颊部血管畸形##糖尿病##脐部感染  Squamous cell carcinoma ##Buccal vascular malformation ##Diabetes ##Umbilical cord infection |

**4.8 Deficiency in Medical Language Generation**

| **Question** | **Answer Points** | **Model Response** |
| --- | --- | --- |
| 任务：假设你是一位专业医生，请根据以下医患对话记录，为患者总结问诊报告。  要求输出格式为：  主诉：“”  现病史：“”  辅助检查：“”  既往史：“”  诊断：“”  建议：“”  问诊对话：  患者：宝宝四个月，最近几天老吐豆腐渣那种。是怎么回事？前几天拉肚子，在吃药  医生：您好  患者：你好  医生：每天几次大便  患者：请问宝宝四个月  医生：咳嗽，流鼻涕，发烧不  患者：最近老吐豆腐渣那种  患者：偶尔咳嗽  患者：不发烧  患者：偶尔流清鼻涕  医生：每天几次大便  患者：这几天都是四五次  医生：宝宝现在是母乳喂养吗  医生：吃奶好不  患者：恩母乳  患者：好  医生：精神状态怎么样  患者：好  医生：现在用什么药呢  患者：金益贝康，和蒙脱石  医生：化验大便没  患者：还有一些小颗白药  患者：没那  医生：腹泻几天了  患者：有四五天了  医生：这种情况建议去医院化验大便看看排除肠炎  TASK: Assuming you are a medical professional, summarize the consultation report for the patient based on the following transcript of the doctor-patient conversation.  The required output format is:  Chief complaint:"”  Present medical history:""  Ancillary examination:""  Past history:""  Diagnosis: ""  Recommendations:""  Interview dialog:  PATIENT: The baby is four months old and has been spitting up the tofu crumb kind for the last few days. What's going on? He had diarrhea a few days ago and was on medication  Doctor: Hello  Patient: Hello  Doctor：How many times a day  Patient: Your baby is four months old.  Doctor：cough, runny nose, fever not  Patient: Recently, he has been vomiting  Patient: occasional cough  Patient: No fever  Patient: occasional runny nose  Doctor：How many times a day  Patient: 4 or 5 times in the past few days  Doctor: Is your baby breastfeeding?  Doctor：How is breastfeeding?  Patient: Yes, breast milk  Patient: Yes  Doctor：How is your mental state?  Patient: Good  Doctor：What kind of medicine are you using now?  Patient：Golden Ibuprofen and Montelukast  Doctor：Have you had any stool tests?  Patient: And some small white pills  Patient: No  Doctor：How many days have you had diarrhea?  Patient: four or five days  Doctor：In this case, it is recommended to go to the hospital for a stool test to rule out enteritis. | {"主诉": ["呕吐", "咳嗽", "流清涕"], "现病史": ["呕吐", "豆腐渣样", "咳嗽", "流清涕", "金益贝康", "蒙脱石"], "诊断": ["肠炎"], "建议": ["医院", "化验"]}  {"complaints": ["vomiting", "cough", "runny nose"], "medical history": ["vomiting", "tofu scum-like", "cough", "runny nose", "Gold Ibuprofen", “montelukast"],“diagnosis":["enterocolitis"], "advice": ["hospital", "laboratory"]} | 主诉：“宝宝四个月，最近几天老吐豆腐渣那种，偶尔咳嗽，不发烧，偶尔流清鼻涕。”  现病史：“患儿四个月大，近期出现呕吐（豆腐渣样），偶尔咳嗽，无发烧，偶尔流清鼻涕，近四五天大便四五次，目前正在服用金益贝康、蒙脱石和未说明名称的小颗白药。”  辅助检查：“未进行大便化验。”  既往史：“无特殊既往病史。”  诊断：“怀疑婴儿胃肠道疾病，需排除肠炎。”  建议：“建议去医院进行大便化验，以排除肠炎。继续母乳喂养，观察宝宝的精神状态和吃奶情况，注意宝宝的脱水症状。”  Complaint: "The baby is four months old, the last few days old vomiting tofu dregs kind, occasional cough, no fever, occasional runny nose."  Present Medical History: "The child is four months old and has recently developed vomiting (tofu dregs kind), occasional cough, no fever, occasional runny nose, four or five bowel movements in the last four or five days, and is currently taking Gold Ibuprofen, Montelukast, and unspecified name of a small white pill."  SUPPORTING EXAMINATION: "No stool tests were performed."  PAST HISTORY: "No specific past medical history."  DIAGNOSIS: "Gastrointestinal disease is suspected in the infant and enterocolitis needs to be ruled out."  RECOMMENDATION: "It is recommended to go to the hospital for a stool test to rule out enteritis. Continue breastfeeding, observe the baby's mental status and milk intake, and watch for signs of dehydration." |

**Appendix B**

**Table S5.** Details of the evaluated LLMs

| **Model Name** | **Organization/Team** | **Version** | **Parameters** | **Access Method / API Platform Link** |
| --- | --- | --- | --- | --- |
| AntAngelMed | Ant Group | / | 80B | Hugging Face (https://huggingface.co/MedAIBase/AntAngelMed) |
| Citrus-2.0 | JD.com, Inc | Citrus-2.0 | 81B | GitHub (https://github.com/jdh-algo/Citrus) |
| INF-Med | InfiX.ai | / | N/A | Proprietary; Developer submission to MedBench |
| WHU_Med | School of Computer Science, Wuhan University | / | 14B | Proprietary; Developer submission to MedBench |
| zhuomuniao-Med | Sinohealth Digital Technology | Guangdong-ZhuoMuNiao | 14B | Proprietary; Developer submission to MedBench |
| TeleChat2 | China Telecom Yikang Technology | / | 14B | Proprietary; Developer submission to MedBench |
| hunyuan-med | Tencent | Hunyuan-MoE | 70B | GitHub (https://github.com/Tencent-Hunyuan) |
| UNI-GPT | Unisound | UniGPT-Med-U1 | 13B | Proprietary; Developer submission to MedBench |
| fusiontech-Med | Fusion Technology | Fusion2-Chat-32B | 32B | Proprietary; Developer submission to MedBench |
| GPT-4 | OpenAI | gpt-4-0613 | N/A | Official API (https://platform.openai.com) |

**Table S6. Prompt Templates Used in MedBench**

| **Type** | **Prompt** |
| --- | --- |
| single-choice question | 任务：请运用专业医学知识回答以下单项选择题。选择正确选项，并将对应的字母填入尖括号 < > 中。  要求：  1.您必须将最终选择的选项字母用尖括号 < > 括起来（例如，< A >、< B >）。  2.选项字母区分大小写。  3.请直接提供最终答案，不要添加任何冗余的文字或解释。  示例：  问题：前囟的正确测量方法是：  A.对角顶点连线  B.邻角顶点连线  C.对边中点连线  D.周径长度  E.邻边中点连线  答案：<C>  Task: Please answer the following single-choice question using professional medical knowledge. Select the correct option and fill its corresponding letter into angle brackets < >.  Requirements:  1.You must enclose your final selected option letter within angle brackets (e.g., <A>, <B>).  2.The option letter is case-sensitive.  3.Provide the final answer directly. Do not output any redundant text or explanations.  Task Example:  Question: The correct method for measuring the anterior fontanelle is:  A. Connecting opposite vertices  B. Connecting adjacent vertices  C. Connecting the midpoints of opposite sides  D. Circumference  E. Connecting the midpoints of adjacent sides  Answer: <C> |
| multiple-choice question | 任务：请运用专业医学知识回答以下多项选择题。选择正确选项，并将对应的字母填入尖括号 < > 中。  要求：  您必须将最终选择的选项字母用尖括号 < > 括起来（例如，< ABC >、< BE >）。  选项字母区分大小写。  请直接提供最终答案，不要添加任何冗余的文字或解释。  示例：自发性气胸常用的临床分型是（）  A．特发性气胸  B．闭合性气胸  C．医源性气胸  D．张力性气胸  E．交通性气胸  Task: Please answer the following multiple-choice question using professional medical knowledge. Select the correct option and fill its corresponding letter into angle brackets < >.  Requirements:  1.You must enclose your final selected option letter within angle brackets (e.g., <ABC>, <BE>).  2.The option letter is case-sensitive.  3.Provide the final answer directly. Do not output any redundant text or explanations.  Example: The commonly used clinical classification of spontaneous pneumothorax is ( )  A. Idiopathic pneumothorax  B. Closed pneumothorax  C. Iatrogenic pneumothorax  D. Tension pneumothorax  E. Communicating pneumothorax |
| open-ended responses question | **Template for Knowledge Q&A / Clinical diagnosis and treatment:**  任务：你是一位资深的医学领域专家。请运用专业的医学知识，准确、全面地回答以下开放性问题。  示例：  问题：体检时，血压测量时的正常范围是多少？  答案：理想血压(BP) 收缩压<120mmHg且舒张压<80mmHg。 未使用降压药物的情况下,非同日3次血压测量收缩压≥140mmHg和(或)舒张压≥90mmHg,可诊断为高血压。血压偏高是指收缩压120~139mmHg和/或舒张压80~89mmHg。  Task: You are a senior expert in the medical field. Please use professional medical knowledge to provide an accurate and comprehensive answer to the following open-ended question.  Example:  Question: What is the normal range for blood pressure measurement during a physical examination?  Answer: Ideal blood pressure (BP) is systolic blood pressure <120 mmHg and diastolic blood pressure <80 mmHg. Without using antihypertensive medication, a systolic blood pressure ≥140 mmHg and/or a diastolic blood pressure ≥90 mmHg on three separate days can be diagnosed as hypertension. High blood pressure is defined as systolic blood pressure 120-139 mmHg and/or diastolic blood pressure 80-89 mmHg.  **Template for Medical Entity Extraction:**  任务：你是一位医学文本分析专家。请根据具体要求，从以下文本中提取特定的医学实体。仅输出提取出的实体，并用逗号分隔，不包含任何额外的对话文本。  示例：  问题：体温36.3℃，呼吸频率19次/min，血压159/98mmHg，神情痛苦，心肺检查无异常，腹平软，全腹压痛，无反跳痛，未触及明显肿大肝脾，肠鸣音6次/min，双下肢无水肿。  需要提取的实体字段  体温(℃)、收缩压(mmHg)、舒张压(mmHg)、呼吸(次/分)、神情状态、心肺检查结果、是否全腹压痛、是否腹部反跳痛、肝脾是否触及、肠鸣音(次/分)  答案：体温(℃):36.3,收缩压(mmHg):159,舒张压(mmHg):98,呼吸(次/分):19,神情状态:痛苦,心肺检查结果:无异常,是否全腹压痛:是,是否腹部反跳痛:否,肝脾是否触及:未触及明显肿大,肠鸣音(次/分):6。  Task: You are a medical text analysis expert. Please extract the specific medical entities from the following text based on the specific requirement. Output only the extracted entities, separated by commas, without any additional conversational text.  Example:  Question: The patient's temperature is 36.3℃, respiratory rate is 19 breaths/min, blood pressure is 159/98 mmHg, and the patient appears distressed. Cardiopulmonary examination is unremarkable. The abdomen is soft and flat, with tenderness throughout, but no rebound tenderness. No significant hepatosplenomegaly is palpable. Bowel sounds are 6 times/min. There is no edema in the lower extremities.  Entity fields to be extracted: Temperature (℃), Systolic blood pressure (mmHg), Diastolic blood pressure (mmHg), Respiratory rate (breaths/min), Visual state, Cardiopulmonary examination results, Abdominal tenderness, Rebound tenderness, Palpable liver and spleen, Bowel sounds (breaths/min)  Answer: Body temperature (°C): 36.3, Systolic blood pressure (mmHg): 159, Diastolic blood pressure (mmHg): 98, Respiration (breaths/min): 19, Mental state: in pain, Cardiopulmonary examination results: no abnormalities, Abdominal tenderness: yes, Rebound tenderness: no, Liver and spleen palpable: not palpable, bowel sounds (breaths/min): 6.  **Template for Medical Record Generation:**  任务：你是一位资深的医学领域专家。请根据所提供的患者症状和病史，生成一份标准化的结构化临床病历。病历必须包含主诉、现病史、既往史和体格检查。  示例：  问题：医生：您好，请坐。哪里不舒服？  患者：医生，我 1 周前体检做腹部 B 超，说我胆囊里有个息肉，当时医生没说具体多大，只说目前没看到结石。这几天偶尔会觉得右上腹有点隐隐的不舒服，但不疼，也没有恶心、呕吐，吃饭、睡觉都正常，就是担心息肉会变大，想再来确认下情况。  医生：那体检报告里有没有提息肉的数量？平时有没有得过胆囊炎、胆结石，或者长期吃什么药呢？  患者：报告里就说有单个息肉。我以前没得过胆囊相关的病，也没长期吃药，平时身体都还挺好的。  医生：您以前有没有对什么药物过敏？家里人比如父母、兄弟姐妹，有没有得过胆囊方面的疾病呀？  患者：我没有药物过敏史，我妈身体一直挺好的，我爸也没得过胆囊相关的病，家里人都没这方面问题。  医生：好的，我先给您做个基础体格检查。测了下体温是 37.0℃，心肺听诊没发现异常，腹部摸起来是平软的，按压右上腹没有压痛，也没摸到肿块，其他部位检查也没发现明显问题。  根据上述医患对话，生成简要的门诊病历，字段包含主诉、现病史、既往史、体格检查内容。  答案：主诉：体检发现胆囊息肉 1 周，伴偶发右上腹隐痛。 现病史：患者 1 周前体检行腹部 B 超检查提示胆囊息肉（单个，未提示具体大小），无胆囊结石。近 1 周偶发右上腹隐痛，无恶心、呕吐，无腹胀、腹泻，饮食、睡眠正常，未行任何治疗。因担忧息肉进展前来复查。 既往史：否认胆囊炎、胆结石等胆囊相关疾病史，否认长期服药史，否认药物过敏史。家族史：父母体健，无胆囊疾病史。 体格检查：T37.0℃；心肺听诊未见明显异常；腹部平软，右上腹无压痛，未触及肿块；其余部位体格检查未见明显异常。  Task: You are a seasoned expert in the medical field. Based on the provided patient symptoms and medical history, generate a standardized, structured clinical medical record. The record must include the chief complaint, present illness, past medical history, and physical examination.  Example:  Question: Doctor: Hello, please have a seat. Where does it hurt?  Patient: Doctor, I had an abdominal ultrasound a week ago during a physical exam. They said I have a polyp in my gallbladder. The doctor didn't say how big it was, only that they didn't see any stones. These past few days, I've occasionally felt a slight discomfort in my upper right abdomen, but it's not painful. I haven't experienced nausea or vomiting, and my eating and sleeping are normal. I'm just worried the polyp might grow larger, so I wanted to come back to confirm.  Doctor: Did the physical exam report mention the number of polyps? Have you ever had cholecystitis, gallstones, or taken any medications regularly?  Patient: The report only said there was a single polyp. I've never had any gallbladder-related illnesses before, and I haven't taken any medications regularly. I'm generally quite healthy.  Doctor: Have you ever been allergic to any medications? Have any family members, such as your parents or siblings, had gallbladder-related illnesses?  Patient: I have no history of drug allergies. My mother has always been healthy, and my father has never had any gallbladder-related illnesses. No one in my family has had any problems with this. Doctor: Okay, let me perform a basic physical examination first. Your temperature is 37.0℃. Auscultation of your heart and lungs revealed no abnormalities. Your abdomen feels soft and flat; there is no tenderness or palpable mass upon pressing your right upper quadrant. Other areas of your body were also examined without any obvious problems.  Based on the above doctor-patient dialogue, a brief outpatient medical record is generated, including fields such as chief complaint, present illness, past medical history, and physical examination details.  Answer: Chief complaint: A gallbladder polyp was discovered during a physical examination one week ago, accompanied by occasional dull pain in the right upper quadrant. Present illness: One week ago, an abdominal ultrasound during a physical examination revealed a single gallbladder polyp (size not specified), with no gallstones. For the past week, the patient has experienced occasional dull pain in the right upper quadrant, without nausea, vomiting, abdominal distension, or diarrhea. Appetite and sleep are normal, and no treatment has been received. The patient is seeking a follow-up examination due to concerns about polyp progression. Past medical history: The patient denies any history of cholecystitis, gallstones, or other gallbladder-related diseases, denies any history of long-term medication use, and denies any drug allergies. Family history: Parents are healthy and have no history of gallbladder disease. Physical examination: Temperature 37.0℃; no obvious abnormalities were found on auscultation of the heart and lungs; the abdomen is soft and flat, with no tenderness in the right upper quadrant and no palpable mass; no obvious abnormalities were found in other areas of the body. |
